# Negotiating belonging: a constructivist grounded theory of transfer students’ experiences in STEM

**DOI:** 10.1186/s40594-026-00633-y

**Published:** 2026-07-12

**Authors:** Katya Hernández Holliday, Saya Shahoy, Stanley M. Lo, Ashley L. Juavinett

**Affiliations:** University of California, San Diego, La Jolla, USA

**Keywords:** Community college transfer students, STEM education, Sense of belonging, Non-traditional students, Neuroscience education

## Abstract

**Background:**

A sense of belonging has been identified as a crucial predictor of persistence in undergraduate degrees. This construct is especially significant within the chilly environment of STEM fields since students’ sense of belonging and recognition as rightful members of the academic community ultimately shape their educational trajectories. However, it has rarely been studied among community college (CC) transfer students attending highly selective universities. This population is of interest because CCs enroll students from underrepresented backgrounds at higher rates than four-year institutions, making transfer students a critical component in efforts to diversify the STEM workforce. Additionally, these students often face unprecedented challenges as compared to their “traditional” peers. We addressed this research gap by conducting semi-structured qualitative interviews with thirteen community college transfer students one and two years after transferring. Analyses applied constructivist grounded theory methodology to examine how these students described a sense of belonging and what experiences they drew from to assess their level of belonging in academic environments.

**Results:**

Transfer students described their sense of belonging as a negotiated process involving internal motivators, values and perceptions, and the messaging received from external institutional, familial, and social factors. Particularly, participants’ experiences of belonging were first marked by an orientation toward building it with some more concerned about their level of fit than others.

**Conclusion:**

By theorizing belonging as a negotiated process, this study shows how community college transfer students in STEM actively interpret academic environments, drawing from both internal motivation and external signals to negotiate a sense of belonging, with students’ orientations toward belonging and internal alignment comprising a major contributing paradigm in the process. This work emphasizes the need to include opportunities for disciplinary exploration in transfer students’ academic trajectories. Potential implications include the design of transfer pathways and advising structures that recognize belonging as dynamic, supporting students’ identity work as central to academic success.

## Introduction

Persistence in science, technology, engineering, and mathematics (STEM) academic programs has long been a focus of research in higher education, given these programs’ importance for workforce development, scientific innovation, and equitable access to high-demand careers. This body of work has identified contributing factors on multiple levels. On the academic level, prior preparation (Radunzel et al., 2016), self-efficacy (Kwon et al., 2023), and academic performance are commonly cited as key predictors of persistence. At the same time, studies have shown the importance of access to social networks and interpersonal relationships including faculty mentorship, academic support and involvement in research experiences for success in STEM pathways (Astin, 1999; Barker et al., 2023). While these studies highlight academic, social, and institutional factors that contribute to persistence, more work is needed to understand how these experiences come together to shape diverse students’ sense of belonging and how belonging relates to persistence in STEM, where inequities in access and preparation persist.

A growing body of research highlights the role of belonging in shaping persistence by emphasizing social and structural influences such as institutional and classroom climates (Duffin et al., 2019; Flores et al., 2024), peer relationships, representation, and perceptions of inclusion (Lewis et al., 2017a, b; Strayhorn, 2019). Despite this work, academic effort, success, and subsequent persistence is often framed as an individual choice, placing responsibility on students rather than on the conditions that shape their experiences (e.g., Duckworth et al., 2007; Park et al., 2020a, b). However, meta-analytic reviews explicitly challenge this individualistic interpretation, demonstrating that internal ‘grit’ is a weak predictor of academic success when isolated from students’ environments (Credé et al., 2017).

Tinto (2015) emphasized the mediating role of motivation and belonging on student persistence, noting that both are “enhanced or diminished by student experiences in college” (p. 2). Importantly, students’ experiences are not uniform nor neutral. Students interpret institutional, academic, and social experiences through their social identities, cultural backgrounds, and prior experiences, which shape their understanding of what it means to belong.

Among these experiences, relational and classroom interactions are especially important. Studies demonstrate that the quality of student-faculty interactions strongly predicts persistence (Christe 2013; Park et al., 2020a, b; Pascarella & Terenzini, 1980) with more recent work showing that students’ satisfaction with the overall college experience mediates this relationship (Loes et al., 2024). Furthermore, Wilson et al. (2015) found belonging at the course level to be more closely tied to engagement than belonging at the institutional level, yet poor instructional practices in STEM can undermine both engagement and belonging (Flores et al., 2024; Seymour & Hunter, 2019). Together, this work highlights the significance of social interactions, classroom cultures, student engagement, and teaching quality in shaping student experiences and supporting STEM persistence.

Yet despite a substantial history of investigation, the literature on undergraduate students’ sense of belonging remains largely focused on individual and classroom-level dynamics within the context of four-year institutions among traditionally aged college students. This narrow focus limits our understanding of how belonging influences persistence among underrepresented students in STEM, including community college (CC) transfer students. Even fewer studies trace students’ trajectories across institutional contexts to examine how a sense of belonging evolves after students transition from 2-year to 4-year institutions.

CC transfer students represent a significant yet understudied segment of the undergraduate population. In the United States, community colleges are two-year public institutions that award two-year associate degrees and serve as access points to higher education. Many students complete their first two years of undergraduate study at a CC before transferring to a four-year university to complete a bachelor’s degree; according to the Community College Research Center, CC transfer students comprised approximately one fifth of all students entering public four-year institutions in the U.S. in 2015 (Velasco et al., 2024). In California, the setting of this study, this proportion increases to nearly one quarter, reflecting the scale and diversity of the transfer student population.

The CC context is particularly important to consider because community colleges disproportionately serve students from historically marginalized and underrepresented backgrounds, including returning and first-generation students as well as low-income and racially minoritized populations (NASEM, 2016). Consequently, the transfer pathway plays a key role in expanding access to four-year degrees and diversifying the STEM workforce (Martinez & Munsch, 2020). Yet despite their growing presence, transfer students frequently encounter structural barriers in four-year institutions including transfer shock (Laanan, 2001), inconsistent advising (Wang, 2016), limited recognition (Jain et al., 2011), and disconnection from campus communities (Gray et al., 2022). These challenges can undermine students’ sense of belonging and persistence in STEM, underscoring the need for research that examines how belonging is negotiated and reconstructed across institutional contexts (Jain et al., 2011; Velasco et al., 2024). Investigating how CC transfer students navigate belonging in STEM can therefore offer important insights into persistence pathways that are often overlooked in research that centers traditional four-year trajectories.

We emphasize that the barriers that CC transfer students encounter are not individual adjustment challenges. They are often manifestations of broader structural inequities which can be understood as an “education debt,” which Ladson-Billings (2006) describes as the cumulative effects of historical and structural inequities that constrain educational opportunity and outcomes. From this perspective, transfer students’ experiences of inconsistent advising, limited recognition, and disconnection from campus communities reflect longstanding systemic inequities that unequally constrain access to resources, recognition, and belonging within higher education. The accumulation of this debt affects students long before they arrive at their transfer institutions and continues to shape their opportunities for inclusion and belonging within STEM. Yet research rarely examines how belonging develops across institutions for students navigating these layered inequities.

Against the backdrop of accumulated educational inequities, we investigate how a diverse group of community college transfer students negotiate belonging among their traditionally enrolled peers (Deil-Amen, 2015) within a highly selective university (Rubin, 2014). Given the role of belonging in persistence (Bentrim & Henning, 2023; Hausmann et al., 2009), motivation (Pedler et al., 2021), and achievement (Knekta et al., 2020), examining how this construct is experienced and interpreted by those navigating complex educational pathways is essential.

Applying a constructivist grounded theory approach (Charmaz, 2014), we extend current understandings by investigating how transfer students interested in neuroscience pathways navigate transitions across institutional contexts while formulating their own conceptions of belonging. Understanding how these students viewed belonging offers insights into the sociocultural and disciplinary challenges specific to both the transfer experience and competitive STEM environments more broadly.

This work began with an understanding of belonging as a perceived ability to occupy academic spaces, engage in meaningful interactions with others and to feel recognized, supported, and valued within the academic community. This includes a sense of comfort, social connectedness, and the freedom to express oneself without fear of exclusion or judgement. However, as data collection and analysis progressed, this working definition evolved in response to students’ own conceptualizations, which emphasized dimensions not fully captured by existing frameworks. Accordingly, we address:

RQ1: How do community college transfer students describe a sense of belonging?
RQ2: What experiences do community college transfer students draw from to assess their sense of belonging in academic contexts?

## Literature review: what is a sense of belonging?

To situate our investigation, we first review broader conceptualizations of belonging that can provide insight into how belonging is experienced, developed, and maintained or shifted over time. We integrate these perspectives with research on belonging in STEM to provide a foundation for understanding the experiences of community college transfer students pursuing neuroscience pathways.

Definitions of belonging vary across disciplines, yet they consistently center on feelings of comfort, support, social connection, acceptance, and inclusion (Hurtado & Carter, 1997; Johnson et al., 2007; Lewis & Hodges, 2015; Wilson et al., 2015). Within STEM, education researchers have emphasized that students’ experiences and learning processes are shaped by social factors including sociopolitical context, identity, and power relations (e.g., Carlone & Johnson, 2007; Esmonde, 2016). Accordingly, Stachl and Baranger (2020) described belonging within university STEM departments as the feeling of being accepted and included as a “legitimate member of an academic community” whose presence and contributions are valued (p. 2), aligning with prior framings (e.g., Baumeister & Leary, 1995; Good et al., 2012; Goodenow, 1993; Walton & Cohen, 2007). Similarly, Strayhorn’s (2019) working definition included “the experience of mattering or feeling cared about” (p. 4).These definitions emphasize the importance of emotional support and feelings of psychological safety and legitimacy for cultivating belonging. However, they provide little insight into how belonging may shift across contexts or over time as individuals’ social and institutional environments change.

Belonging is not uniformly experienced nor sustained. Accordingly, Mahar et al. (2013) described a transdisciplinary and multidimensional understanding of belonging encompassing five themes: (1) subjectivity, (2) groundedness, (3) reciprocity, (4) dynamism, and (5) self-determination.

First, a sense of belonging is subjective because it is a personal perception of whether one is valued and respected within a space as well as how well they fit. Importantly, a sense of belonging must be distinguished from social participation because it encompasses individual reactions or qualitative responses to interactions within the group rather than focusing on inclusion alone (Mahar, 2013, p. 1030).

Second, belonging must be anchored or grounded in some referent group. That is, “One belongs *to* something” external (Mahar, 2013, p. 1030). In education, a referent group may be a school, peer group, classroom, or an entire campus community. Therefore, any measures or assessments of belonging require specificity about what individuals’ perceptions are in reference to.

Third, belonging is strengthened by feelings of relatedness or connectedness that are mutually shared between the individual and the group. To contribute to a sense of belonging, these feelings must go beyond shared physical or behavioral characteristics. They must foster feelings of safety and comfort which are often cultivated through shared beliefs, understandings, or experiences.

Fourth, belonging is dynamic. Complex interactions between personal and environmental factors often contribute to or diminish individuals’ sense of belonging. Environmental conditions, social relationships, and individual perceptions of environments and relationships may shift over time, leaving temporary or long-lasting effects on an individual’s sense of belonging. For students, belonging can therefore change as they encounter new institutional contexts, social climates, peer groups, instructional practices, or forms of recognition and exclusion. Barriers such as geographic location, political and economic conditions, prejudice, discrimination, and exclusionary social norms may further impact whether an individual feels that they *can* belong within a particular environment. Thus, dynamism refers to how conditions may change over time and how belonging is continually shaped by interactions among personal perceptions, social relationships, and environments.

This dynamism is captured by Dost’s (2026) exploratory study which shows how students’ understanding of and experiences with belonging change across academic stages. By investigating differences and commonalities between A-level students (typically those in the final two years of secondary school), undergraduates, and post-graduates’ sense of belonging, Dost identified distinct features that comprise sequential phases of belonging: (1) the adaptation phase, (2) the integration phase, (3) the continuum phase, and (4) the transition phase. In the adaptation phase, a sense of belonging is informed by positive interactions with group members, intrinsic motivations, and internal emotions. During the integration phase, students’ sense of belonging is intertwined with feelings of connectedness to the environment as well as collaboration with group members. A strong sense of belonging during this phase is defined by shared beliefs, interests, and common goals; mutual recognition; and strong social bonds. During the continuum phase, a sense of belonging is informed by students’ experiences in the previous two phases and the accumulation of positive or negative experiences over time. In the transition phase, individuals experience shifts in their social or physical environment which prompt engagement in new social relationships or contexts. As they navigate these changes, individuals may identify new referent groups in which to seek belonging, reinitiating movement across the preceding phases.

Fifth, individuals have the right to choose whether or not they want to belong. Yet an individual may have the desire and the supposed qualifications and still be prevented from belonging to a group through discriminatory practices. This nuance highlights that belonging may first be impacted by whether or not an individual feels that they *can *belong and secondarily by whether or not they *want *to belong.

Mahar’s (2013) five-part conceptualization of belonging positions it as a subjective, relational, and context-dependent experience rather than as an individual characteristic. Belonging is grounded in particular groups and environments, shared through reciprocal relationships, and subject to change as students encounter new contexts, barriers, and forms of recognition or exclusion. At the same time, self-determination highlights that belonging arises from both an individual’s desire to belong and their perceptions about whether belonging is attainable. This framing provides a foundation for understanding belonging as a complex, personal process involving multiple internal, relational, and external dimensions.

### Belonging as a contextually situated, psychological construct

Psychological frameworks recognize the foundational role of belonging (Imboden, 2024). Maslow’s (1970) hierarchy of needs theorized belonging as a core human need closely related to motivation. Strayhorn’s (2019) theory of college student belonging extended this perspective, framing belonging as a basic need, a fundamental motive, akin to mattering, and a condition that must be continually satisfied. At the same time, early childhood experiences form the foundation of how people navigate belonging, informing expectations about how similar conditions can be recreated in new environments (Vaccaro & Newman, 2022). Consequently, students enter academic environments with their own ideas of what it means to belong. Their experiences and perceptions of belonging are further shaped by their social and cultural identities and environmental contexts. Thus, although belonging is experienced at the individual level, students’ feelings of connection, acceptance, and fit are influenced by academic, social, and institutional environments. Belonging can therefore be understood as both individually experienced and contextually situated.

Dost’s (2024) study on STEM belonging during the COVID-19 pandemic found that stereotype vulnerability was negatively associated with STEM belonging, with differences observed across gender. The relationship between COVID-19-related stress and sense of belonging also varied by gender, ethnicity, and socioeconomic background. In the post-pandemic context, students emphasized the importance of psychological safety, comfort, and authentic connections to rebuild a sense of belonging in academic settings. Moreover, female, non-binary, and first-generation students described belonging as feeling safe, valued, and heard within inclusive environments. Similarly, Johnson et al. (2007) found that white students reported the highest levels of belonging across majors, while interactions with diverse peers and perceptions of campus racial climate significantly predicted belonging, but only for students identifying as “Hispanic/Latino.” Together, these findings demonstrate that students interpret and experience the same social environments in vastly different ways.

Research has also emphasized the relational nature of belonging by highlighting the important role of student-faculty interactions (Christe, 2013; Park et al., 2020a, b; Pascarella & Terenzini, 1980). Zumbrunn et al. (2014) showed that students who received academic and social support from instructors reported greater feelings of belonging and self-efficacy, which were associated with higher achievement. Relatedly, Wilson et al. (2015) found positive associations between course-level belonging and students’ effort, participation, and emotional engagement in STEM coursework. Yet other studies found that poor instructional practices can undermine engagement and belonging (Flores et al., 2024; Seymour & Hunter, 2019), emphasizing the important role faculty can play in supporting belonging through positive classroom experiences.

However, faculty support is not equally accessible. Undocumented students in the U.S. report numerous obstacles preventing them from engaging with and developing relationships with faculty including feelings of invalidation and concerns over instructors’ views on immigration issues (Diaz-Strong, 2025; Stebleton & Aleixo, 2015). In their study of freshmen across institutions in the United States, Park et al. (2020a, b) found that while Black students reported higher levels of interaction with faculty than other groups, they were also more likely to report racial or ethnic discrimination. While faculty discrimination had the strongest negative effect for Latine^1^ students, discrimination from faculty negatively impacted retention in STEM across all groups, overshadowing the potential benefits of positive interactions. Together, these studies suggest that belonging is shaped through students’ perceptions of interactions with peers, faculty, and institutional climates. They also emphasize that belonging is contextually situated and informed by students’ intersecting identities and institutional cultures.

### Belonging in STEM

In addition to broader social, institutional, and environmental contexts, students must also navigate belonging within disciplinary cultures. In STEM, belonging is shaped by both broader contexts and by disciplinary norms and expectations. 

STEM subjects are often framed as domains of objectivity, detached from sociocultural context (Högström et al., 2025; Lederman et al., 2002). This framing is particularly important for understanding the experiences of historically underrepresented students who are often subjected to disciplinary norms that marginalize their identities, inhibiting full participation in STEM communities (Hansen et al., 2023; Payne et al., 2025).

Students are positioned within STEM communities according to their social identities and the extent to which they are recognized as legitimate participants. For example, Lewis et al. (2017a, b) found that women in physics consistently reported lower levels of belonging than their male counterparts, and that belonging was a stronger predictor of intent to persist in graduate studies for women than for men. In Robnett’s (2015) study, a majority of women reported experiencing gender bias. Gender bias, in turn, was found to be associated with lower STEM self-efficacy.

Furthermore, inequities in belonging and access to support can persist even in institutions with high minority representation. Flores et al. (2024) conducted a study at a higher education institution designated as both an Asian American and Native American Pacific Islander-serving institution (AANAPISI) and as a Hispanic-serving institution (HSI). Despite these designations, Latine and Black students noted a lack of representation within STEM classrooms and exclusionary social practices among their mostly-Asian peers. At the same time, students noted that STEM faculty tended to perpetuate the chilly and competitive culture of STEM classrooms. These findings emphasize that belonging and participation in STEM are shaped by social identities and institutional climates and that exclusionary environments can signal who does and does not fully belong in STEM.

Exclusionary practices in STEM environments are not isolated experiences; they are rooted in longstanding cultural norms within STEM disciplines. In their landmark paper, Seymour and Hewitt (1997) argued that STEM culture embraces a “weeding out” approach. Since this work, many programs have broadened their perspectives of students’ potential and have dissolved some overt “weed out” practices. Nonetheless, many structures, policies, and mindsets that reflect the weed-out culture remain (Carpenter, 2024; Seymour & Hunter, 2019; Weston et al., 2019). This challenge is often intensified for those navigating transfer pathways and is especially damaging to students from historically marginalized backgrounds, including women, students of color, and first-generation college students and can undermine their sense of belonging and legitimacy within STEM communities.

### STEM transfer students and sense of belonging

Transfer students in STEM face layered challenges as they must contend with institutional processes associated with transfer pathways in addition to the aforementioned exclusionary disciplinary norms. In one study of female transfer students in computer science and physics, participants described challenges related to credit transfer, adjusting to the academic rigor of STEM coursework, and developing a sense of belonging. Findings suggested that navigating these layered challenges required access to information, confidence, and forms of privilege that are not equally available to all students, further emphasizing how institutional processes and policies can contribute to feelings of exclusion (Steele et al., 2020). Allen et al. (2022) demonstrated how belonging is institutionally shaped through their study of Black women in STEM who transferred from community colleges. Their participants described community colleges as more supportive than university environments and reported instances of racism, sexism, and difficulty connecting with faculty, creating a sense of alienation within the university context.

These challenges are often compounded by assumptions about academic inferiority (Wood & Moore, 2014), limited access to research opportunities, and unfamiliarity with the “hidden curriculum” of higher education (Jackson, 1990; Schudde & Jabbar, 2024). After transfer, students often face institutional cultures that overlook or undervalue their lived experiences. For example, Jain et al. (2011) describe how transfer students are rendered invisible through exclusion from targeted programming and dominant student narratives. Exclusionary practices, combined with ineffective or limited advising (Steele et al., 2020; Wang, 2016) and the separation from familiar peer communities, can exacerbate feelings of isolation. As a result, CC transfer students must grapple with belonging within environments that were not designed with them in mind. These factors can position students as “behind,” a deficit view that can restrict access to mentorship and discourage participation. Because STEM environments frequently equate merit with legitimacy, this positioning can be deeply damaging to students’ sense of belonging as their presence and capabilities may be continually contested.

Although Chen and Liu (2026) focused on how students developed a STEM identity rather than belonging, their findings are relevant to this discussion since students’ self-perceptions, feelings of legitimacy, and experiences of recognition are closely related to their sense of belonging. Through their quantitative analyses, Chen and Liu found that STEM interest, STEM recognition, and post-transfer social adjustment were significantly positive predictors of STEM identity. These findings suggest that belonging is not only determined by whether students gain access to institutional resources and spaces but also by whether institutional environments support their social adjustment and recognition as legitimate members of STEM communities.

Acknowledging the role of institutional environments in shaping students’ social adjustment, some institutions have introduced programs designed to support students from marginalized communities, including low-income students, women, and CC transfer students. LaDue et al. (2024), for example, found that low-income, high-achieving STEM students often struggled with academic pressure, feelings of isolation, and unsupportive relationships. Though these challenges can negatively impact students’ sense of belonging, those who participated in a near-peer social support program developed meaningful connections and engaged in discussions that normalized their struggles. These experiences, in turn, helped students strengthen their sense of belonging in STEM.

While existing research affirms the importance of belonging in higher education, it often conceptualizes it as a static experience bound to the institution. This characterization frames belonging as a single-site experience that does not capture the experiences of students who move across educational contexts, such as CC transfer students. Much of the research focuses on four-year institutions, typically assuming first-time college students as the norm. Additionally, studies that rely on survey data often measure belonging at a single point in time, treating belonging as fixed and individually constructed. Although some scholars acknowledge that belonging is a dynamic and relational process, many researchers still frame it as inevitably shaped by institutional factors, peer relationships, and achievement (Tinto, 2023), overlooking students’ individual interpretations and negotiations. As a result, these frameworks underemphasize how students make meaning of their experiences to assess whether and where they belong.

This study builds on prior scholarship by shifting the frame from belonging as a static, institutionally defined outcome to belonging as an evolving, negotiated process. We center students’ own meaning-making as they navigate multiple educational settings, allowing for a more nuanced understanding of how belonging operates across transitions. In doing so, we extend theoretical frameworks of belonging beyond single-site, four-year contexts.

## Methods

### Participants

Participants included undergraduates recruited from a program designed to support CC transfer students in neuroscience-related fields. For a more detailed description of the support program, please see Zuckerman et al. (2022). In the U.S., community colleges are 2-year institutions from which students can transfer to 4-year universities to complete bachelor’s degrees. Students joined the program the summer prior to transferring and received support for two subsequent years. All participants attended the same research-intensive, doctoral-granting university, classified as having very high research activity within the U.S. Carnegie Classification system (Carnegie Classification, 2023). The program provided students with a stipend to promote engagement in lab research, assigned faculty mentors, and hosted quarterly social events. Although involvement in this program was shared among the students, the support of the program was not a focus of the interviews as we aimed to center on factors shaping students’ sense of belonging without leading their responses (see *Data Collection*).

Thirteen students agreed to participate in the study. Seven were enrolled in their first year at the transfer university (third-year undergraduates) while six were in their second year (fourth-year undergraduates) at the time of interview. Neuroscience was selected as the disciplinary site for this study because it represents a cross-disciplinary STEM field that draws on multiple domains including biology, chemistry, psychology, mathematics, and computer science. Its interdisciplinary and research-intensive nature makes neuroscience a strong case for understanding persistence and belonging within competitive STEM environments more broadly.

Participant majors included: Biochemistry (1), Cognitive and behavioral Neuroscience (2), Clinical Psychology (2), Cognitive Psychology (1), Cognitive Science (1), Cognitive Science with specialization in Neuroscience (1), Microbiology (1), Neurobiology (3), and Psychology (1). One student did not report their major.

### Ethical considerations

This study was approved by the Institutional Review Board at the University of California, San Diego. All participants provided informed consent prior to their participation. Participation in the study was voluntary and did not affect students’ standing in the support program nor in their courses. Although the third and fourth authors were directly affiliated with the program, they did not conduct interviews, access identifiable data, or participate in data analysis or interpretation to mitigate potential power dynamics. Participants were assigned pseudonyms and identifying details were removed to protect confidentiality.

The first author, who conducted all interviews and primary analysis, and the second author, who assisted with analysis and interpretation, were not affiliated with the program and held no evaluative authority over participants. The first author shares a background as a returning community college transfer student, which facilitated rapport and co-construction of meaning during interviews. Throughout the process, reflexive memoing was used to critically examine how this positionality influenced interpretation.

### Data collection

Audio data were collected through individually conducted semi-structured interviews lasting between 30 and 60 min. These were held via Zoom in the summer, following the conclusion of the Spring academic term. Each conversation focused on participants’ experiences and sense of belonging across three key contexts: their community college (CC), their transfer university, and their field of study at the time of the interview. Interview protocols included open-ended questions designed to elicit reflections on academic, social, and institutional dimensions of belonging. Importantly, the questions were framed broadly rather than tied to participation in the support program. This allowed participants to reflect on belonging beyond program contexts. Table 1 provides representative examples of interview questions.

**Table 1:** Sample questions from the semi-structured interview protocol

Context of belonging	Question
Academic	In what ways do you feel/not feel prepared for a PhD program? Does your sense of belonging in your field affect your desire to pursue a graduate degree?
Social	Please describe an area or context in which you feel a deep sense of belonging.
Institutional (University)	Can you describe a moment when you felt you truly belonged at your university? a. What aspects of your identity influenced this feeling? b. Is there a difference between feeling like you belong within the university versus within your department or field? What would you say it means to belong at your university?
Field-specific	What has made you feel supported to pursue your field? Have you ever felt like you needed to change something about yourself to belong in your field? Can you describe a moment when you felt you truly belonged in your field?

All interviews were recorded and automatically transcribed using Zoom’s transcription feature. Transcripts were then reviewed and corrected for accuracy using MAXQDA, this software was also used to distinguish each speaker and prepare the data for coding and analysis.

### Data analysis

Analysis followed a constructivist grounded theory approach (Charmaz, 2014; Strauss & Corbin, 1994) to center the co-construction of meaning between researcher and participants, while drawing on Strauss’ (1987) systematic coding procedures to support analytic rigor and transparency. While participants’ lived realities were treated as contextually situated and unique, we engaged in systematic comparison to develop interpretive patterns across participant experiences, consistent with Strauss’ emphasis on identifying shared social processes. Throughout, conceptual categories were understood as interpretive constructions emerging from analytic engagement rather than as objective discoveries embedded in the data.

Consistent with grounded theory methodology (Charmaz, 2014), we began with a working definition of belonging informed by the research base. This definition evolved throughout analysis as participant responses reflected personal understandings of belonging. The final conceptualization of belonging, expressed in the findings, combines both initial framing and emergent insights grounded in participant experiences.

Analyses involved the constant comparative method (Charmaz, 2014; Strauss & Corbin, 1994) as data were iteratively compared within and across interviews to refine emerging categories. Following each interview, the first author produced summaries, noting events or experiences related to concepts matching the working definition of belonging and developing additional questions that could help us delve deeper into individual conceptions of belonging; these were later used to reconfigure interview protocols as needed.

Initial coding was conducted line-by-line, by the first and second authors, to capture participants’ meanings with attention to context and social actors (e.g., community college or transfer university context, faculty, students, counselors). The purpose of initial coding was to pinpoint contexts and actors that enabled or hindered the cultivation of a sense of belonging. This was followed by focused coding to synthesize and conceptualize the most salient patterns in the data.

As interviews proceeded, earlier transcripts were revisited to continue the constant comparison process, focusing on emergent signals of belonging and building on earlier attention to contexts and interactions. Coded segments across participants were revisited, revised, and grouped into focused codes in later stages to develop conceptual categories.

Following data collection, the first author conducted a second round of coding to identify and compare instances that related to students’ feelings of belonging. Together, the authors established conceptual codes when patterns were evident. Additional descriptive codes emerged where meanings remained uncertain. This stage of analysis focused on finding evidence to determine assumptions shaping participants’ understandings of what it means to belong in academic settings. The constant comparison across participants served to interrogate assumptions by allowing each theme to prove relevant through its continued presence in the data while memoing supported reflexive engagement with the data and analytic transparency across researchers.

A subsequent round of axial coding, adapted from Strauss (1987), examined relationships between conceptual categories to construct themes while maintaining a constructivist orientation to interpretation. During this stage, descriptive codes which housed 3 or fewer segments were either dropped, absorbed into related categories as subcategories, or considered as dimensions of a larger category. Additionally, during this stage, the team developed a detailed codebook, refining the definitions and dimensions of each conceptual category grounded in participant experiences. The goal was to ensure that each category captured patterns across multiple participants, by including quotes from multiple students within the codebook, supporting the development of a robust and fitting grounded framework. This process resulted in a total of 5 core conceptual themes encompassing categories related to students’ beliefs about belonging (see Table 2).

A fourth analytical pass aimed to refine categories and identify disconnects between student experiences and conceptual labels. During this stage, we focused on drawing connections between larger categories, working toward developing a conceptual model of the data. This was followed by selective coding, which focused on finding a central category that encapsulated how participants experienced a sense of belonging.

**Table 2:** Conceptual themes and related categories for a theory of belonging as a negotiated process

Themes	Categories	Definitions	Exemplars
Orientation toward belonging	Unaware orientation	A view of belonging as consistently available and, therefore, unnecessary to pursue	*“Everybody has been very supportive and very willing to…let me do what I want…”*
	Disinterested orientation	Choosing not to engage with or prioritize building a sense of belonging in academic environments	*“I couldn’t say I feel involved in the larger community here…that’s probably also by choice*,* I’m kind of more micro focused on what I’m trying to do than having the college experience.”*
	Conditional orientation	Belonging is conditional on performance or adherence to social norms and expectations	*“I feel like I have to be mindful of how much personality I show because I feel like there’s people who can show their full personality and be perceived as charismatic but I don’t know if I can do that. So I’d rather be perceived as quiet.”*
	Authenticity orientation	True belonging allows authentic engagement	*“[To belong] I should be able to not have to code switch too hard in the space. I should not have to change my appearance…how I present to be accepted…I don’t feel like I should have to do the corporate jargon robot speak to fit in the space.”*
Motivation	Curiosity and personal interest	Often described as passion, this category describes the intrinsic motivation students’ draw from to continue in their fields	*“I chose neuroscience because I knew this would be a field that*,* even if…things got tough*,* that curiosity would help propel me to keep going…”*
Perceived academic performance	Academic struggle	Feelings of inadequacy stemming from poor grades or challenging material erode belonging	*“But then I wouldn’t understand things or do as well as I’d hope and then I felt like I didn’t belong well because I was like*,* oh*,* I’m not performing well.”*
	Academic success	Success justifies students’ participation in their field	*“The positive reinforcement around performance in school has definitely encouraged me…made me feel like I belong here.”*
	Comparison with peers	Perceptions of peers’ performance are used to assess belonging	*“It might be imposter syndrome*,* but I think that’s one thing I don’t feel like I’m up to par with…the amount of knowledge and expertise that they have.”*
Social dynamics	Social positioning	How students felt positioned among peers within academic spaces often according to race, age, socioeconomic or transfer status	*“I always felt like there’s always still a barrier*,* even when you’re in these groups…I was hoping to make some kind of connection…but it felt very cliquey. ”*
	Social reinforcement	Feedback and social connection (or the absence thereof) that reinforces or challenges students’ belonging	*“I’ve never really felt like the old guy in the room until I got [here] and I realize*,* wow*,* these are people that are so far removed from who I am as a person.”*
	Representation	Institutional cues of belonging including cultural and experiential representation and assumed narratives	*“I look super white*,* but I am full Mexican…if I wanted to blend in with white people and I picked up a bunch of white hobbies*,* like surfing…and I was dressing like surfer…so if I wanted to hang out with the baseball guys or whatever*,* I could but I felt like this disconnect like I don’t really want to…I remember being in those [Psychology club] meetings and being like I could not hang out with these people.”*
	Family dynamics	Family and community expectations that support or challenge students’ desires to belong in academia	*“My parents raised me…to be like*,* ‘yeah*,* you need to be a strong*,* independent woman*,*’ I don’t feel like a strong independent woman going into grad school without some kind of financial savings…some kind of blanket of security.”*
Institutional conditions	Equal expectations under unequal conditions	Students’ realizations that their institution’s universal standards do not account for their lived realities	*“I feel like with transfers you’re expected to be at the same level as a full 4 year…for example*,* with the research lab…you don’t have research experience or limited research experience…even with scholarships they say you have to have a minimum of this many extracurriculars or this many years of research and I feel like that’s not realistic as a transfer.”*
	Structural inequality	Unacknowledged inequality embedded within institutional systems	*“Research wasn’t really built for underrepresented minorities…it’s very obvious with the culture and atmosphere.”*

Note. Italicized text represents verbatim participant quotations.

## Results

### Belonging as a negotiated process

Across participant narratives, belonging did not emerge as a stable feeling or fixed status. Instead, it was experienced as a dynamic and continually shifting process, informed by changing contexts, evolving relationships, and personal reflections. To capture this complexity, findings advance a theory of *belonging as a negotiated process*. This theory captures how students continually interpreted, questioned, and redefined their internal beliefs and external environments to determine the extent to which they belonged. Rather than maintaining a static sense of inclusion, students described ongoing negotiations across academic, social, and institutional contexts. The process unfolded over time as students drew from personal beliefs and values as well as external signals about who is perceived as belonging in academic spaces with each carrying varying weights within varying contexts.

**Fig. 1:**
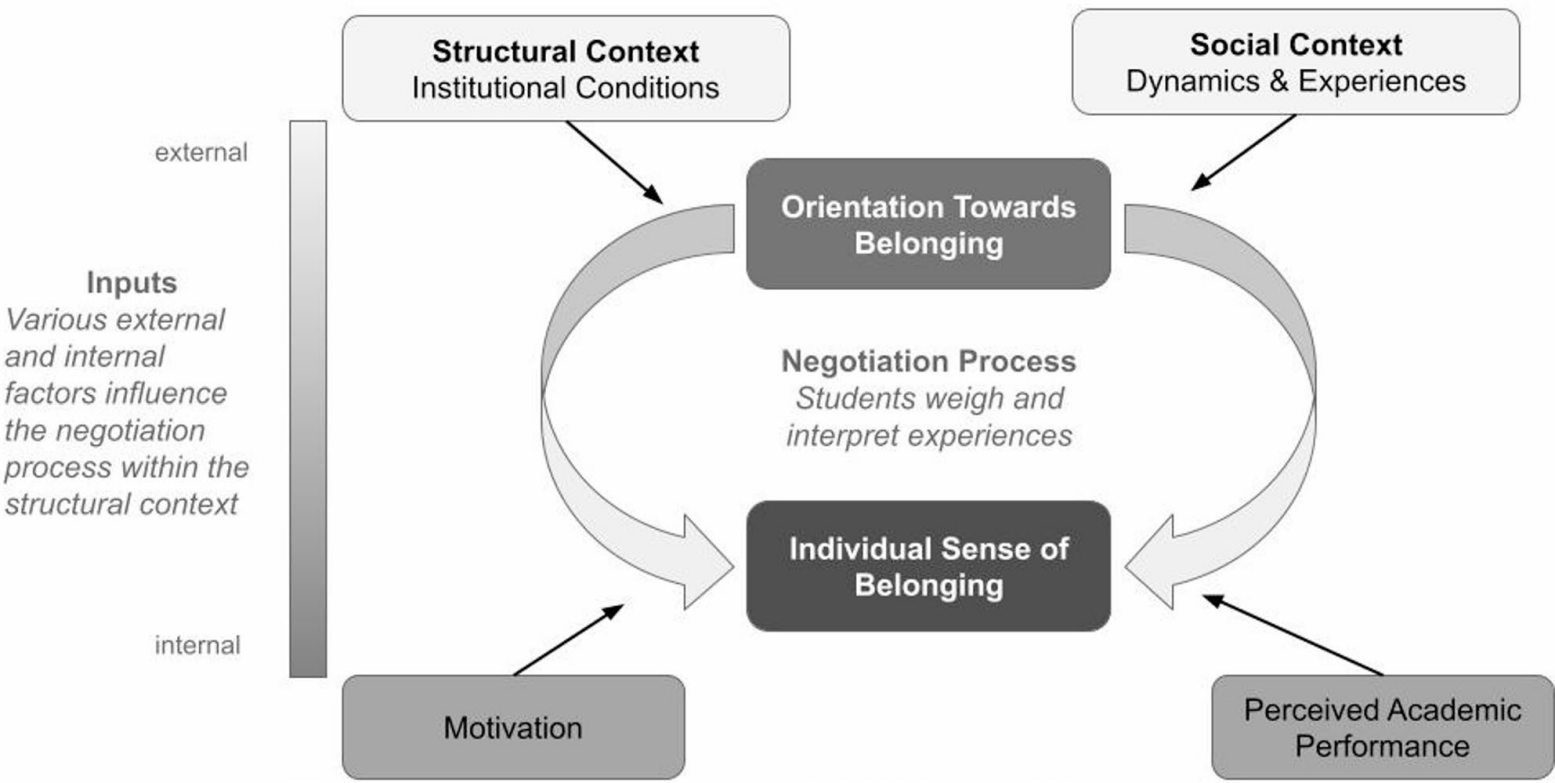
Conceptualization of a sense of belonging as a negotiated process

Participants began negotiations with an initial, context-dependent *orientation toward belonging*. Over time, additional factors influenced how that sense of belonging was sustained as they drew from intrinsic beliefs and values that comprised their *motivation* and interpreted their *perceived academic performance*, including grades and self-efficacy. *Social dynamics* such as how they felt positioned, validated, and represented in academia further informed their assessments of belonging while *institutional conditions* introduced additional layers of complexity. Together, these interrelated themes build a process-oriented model of how students negotiate a sense of belonging (see Fig. 1); they are ordered in the findings according to their proximity to the self.

#### Note

Students’ negotiations of belonging involve internal and external influences which vary individually with some emphasizing or ignoring certain factors. Nonetheless, orientation toward belonging often carried the most weight as it determined whether participants sought to establish a sense of belonging. Meanwhile, perceived academic performance and social dynamics contributed to internalized self-perceptions (e.g., self-efficacy) by drawing from external influences. (e.g., grades, interpersonal relationships/interactions).

### Orientation toward belonging

Students’ *orientations toward belonging* represent the foundational perspectives they bring into academic spaces. Early experiences of belonging inform how it is assessed and pursued within new contexts, guiding how and whether students negotiate belonging according to perceived levels of recognition, inclusion, and participation. Narratives revealed at least five orientations reflecting beliefs about how belonging is formed, earned, or sustained within academic contexts. These orientations vary by perceived levels of inclusivity, individual desire to belong, academic fit, and authenticity.

*Unaware Orientation.* For some, effortlessly maintaining a strong sense of belonging within non-academic contexts may contribute to more detached or pragmatic attitudes. An *unaware orientation* emerged among students who never experienced or perceived barriers to inclusion or comfort in academic spaces. From this perspective, belonging was described as something consistently available and, therefore, unnecessary to pursue: “I have yet to be around somebody who is willing to try and actively put me down, or actively deter me from my goals… everybody has just been very supportive and very willing to…let me do what I want if I want to do it.” This category suggests that, for some, belonging is taken for granted and personal drive or access becomes the primary anchor to their academic and career pursuits, meaning the typical work of negotiating belonging becomes unnecessary or invisible.

*Disinterested Orientation*. Similar to the unaware orientation, other students adopted a *disinterested orientation*, choosing not to engage with or prioritize connection in academic environments. This orientation was especially common at CCs: “I really did feel like I treated community college as much as I show up, I go to class, I get my work done, I leave…I wasn’t there to make friends, especially after failing out of [a 4-year university].” However, this stance continued after transfer as well: “you don’t need a social life. You have things to do. That’s what mentality I came to [the transfer university] with.”

For some, a disinterested orientation was driven by a mission or goal-oriented approach to higher education, where belonging was seen as irrelevant to achieving long-term goals: “I wasn’t actively looking for a sense of belonging…I just kind of viewed it as…this is another thing I have to do to get to the goal…which is again, research, PhD.” Yet despite their limited focus on social connection, many students maintained strong peer networks and progressed into graduate studies or research careers, suggesting that disinterest in belonging does not imply isolation or precede academic withdrawal.

This orientation highlights that negotiation does not always involve an active pursuit of belonging. Students may instead redefine or minimize its importance. By framing education primarily as work rather than a social experience, students negotiated belonging by deemphasizing it. This reframing can transform disinterest into a protective stance that may buffer them against more turbulent or emotionally involved dimensions of belonging.

*Conditional Orientation.* A *conditional orientation* toward belonging emerged among many students but was especially influential for those who consistently perceived themselves as outsiders in academia. These students often expressed belonging as something that applied to others but was unattainable for themselves. For example, one participant described belonging to the institution as: “you’re a STEM major, you’re in the hustle culture.” She added that those who belonged at her transfer university, “take themselves really seriously, and they take their education very seriously. They’re in [the library] 20 hours a day type of thing.” Belonging from this perspective depended on willingness and ability to meet performance expectations, behavioral norms, and institutional standards.

Rather than feeling inherently valued or welcomed, students with a conditional orientation believed that their acceptance had to be continually earned, aligning with a guest positionality (Calabrese Barton & Tan, 2020). This constant need to negotiate legitimacy through performance created a fragile sense of belonging that easily crumbled when expectations were unmet. Over time, the pressure of continuous negotiation led to fatigue, disillusionment, and in some cases, psychological or emotional withdrawal from academics: “it just knocked me off my flow and I’m like, okay maybe I should rethink things more.” However, not all students applied conditionality in a way that implied deficiencies:

*Navigating this school as a Black student*,* the one thing I don’t think anyone could take away from me is the fact that I earned my way into this school. I worked really hard…I know what I’ve done…no one can make me feel like I don’t belong here*,* because I know deep down that I do*,* and that if I didn’t deserve to be here*,* I wouldn’t be here.*

For this student, the implicit standards of academic performance provided justification for their presence. While their sense of belonging was still contingent on performance, the belief that they have already negotiated and met standards reflects a strong internalization of academic abilities. This form of conditional negotiation may boost academic self-efficacy, providing a buffer against external pressures while sustaining motivation and high performance despite challenges.

*Authenticity-focused Orientation*. Opposing a performance-centered view of belonging, students expressed a desire for belonging rooted in freedom of expression. Students with an *authenticity-oriented* view of belonging described feeling most connected in environments where they could bring their whole selves. This orientation did not focus strictly on academic performance, but included norms around behavior, culture, and racial, gendered, or sexual identities. For these students, belonging was deeply tied to self-expression or, negatively, the fear of judgement and pressure to conform. When environments felt unwelcoming of their identities, students described feeling shunned:

*I remember this vividly…I had put in long braids*,* I had put on nails*,* and my lashes on. I had…decided I wasn’t going to code switch as hard as I was in the past. So I was being more authentically Black*,* culturally*,* and I definitely felt a little bit less taken seriously by some of the people that I was working with…you could kind of want to conform to what everybody else looks like and what everybody else is doing just for your comfort but I feel like that’s not specific to neuroscience.*

Students who centered authenticity often expressed shifting orientations towards belonging. On the institutional level, they described conditionality tied to academic performance but on a personal level, they often applied an authenticity-oriented approach. These shifts affirm that belonging is not a fixed experience, but an ongoing negotiation of self-presentation in response to varying levels of inclusion, validation, or threat. The emergence of multiple orientations reveals how students weigh the costs and benefits of authenticity, continuously negotiating how much of themselves to reveal based on perceived expectations, safety, and cultural alignment within a given space.

### Motivation

Orientation towards belonging determined how students approached academic spaces, seeking to earn their place, assuming belonging, or rejecting it altogether. However, their ability to sustain a sense of belonging was dependent on additional factors. Particularly, students often relied on intrinsic motivation to validate their belonging. Here, we define intrinsic motivation as an inherent interest that drives individuals to explore, learn, and seek out challenges (Ryan & Deci, 2000) and encompasses participants’ descriptions of curiosity, passion and personal interest.

Students whose belonging was firmly anchored by intrinsic motivation could not fathom impediments to belonging. When asked to reflect on what made her feel like she did not belong in neuroscience, one student commented: “I don’t understand ‘belong’ in this sentence because if you like that major, and you do your best there, so you belong there.” Others echoed this sentiment, noting that those who belong in the field are simply those who are curious, driven, and passionate about the work.

Intrinsic motivation also served as an anchor, keeping students engaged in their discipline:

*[Finding my courses interesting] helps me stay curious…I think that’s why I chose neuroscience*,* because I knew this would be a field that even if…things got tough*,* that curiosity would help propel me to keep going and not give up as opposed to another field like biology or something.*

This reflection emphasizes the role of intrinsic motivation in students’ academic resilience. Moreover, students viewed their resilience and ability to overcome challenges as indicators of belonging with some arguing that the “ability to do something hard, mess up at it, then get up and try again” aligned well with qualities of individuals who belong in neuroscience. These experiences describe how motivation functioned as a means of negotiation as students leveraged their curiosity and persistence as evidence of belonging, even when external markers of acceptance were absent or conditional. However, internal motivation was not always enough as students grappled with perceptions of acceptable academic performance relative to their peers.

### Perceived academic performance

Students relied on their *perceived academic performance* to gauge their sense of legitimacy within academic spaces. We use legitimacy to refer to a sense of having a rightful place and voice in academic spaces, a perception that was often used to evaluate belonging. Here, performance functioned as a key dimension of negotiation allowing students to assess their standing through both internal benchmarks and perceptions of external evaluations by peers, faculty, and the institution. Negotiations unfolded through three experiences: *academic struggle*,* academic success* and *comparisons with peers*.

*Academic Struggle.* Struggle was not limited to academic performance as measured by grades; it also included emotional and psychosocial dimensions. For example, one student felt “deterred by the field” of neuroscience after encountering lab work and readings that left her feeling lost: “I have no idea about what we’re talking about…maybe I’m not for this, this is way too hard.” Another participant shared, “It’s hard to feel like you belong if you don’t feel like you’re good enough.” These instances demonstrate the high value students place on feeling or being perceived as knowledgeable; when students are unable to achieve or display competence, it can lead to feelings of unworthiness or self-doubt.

Importantly, academic struggle influenced students’ ability to accept support even within environments where they felt otherwise affirmed: “[Not belonging] challenges even that one area where you do feel like you belong.” In this way, academic struggle negatively impacts students’ sense of legitimacy, requiring them to re-negotiate their belonging in the face of failure. For many, unsupported negotiations led to instability, which often inhibited the development of a stable sense of belonging.

*Academic Success.* In contrast, many students drew upon their *academic success* to justify their place in the field. For these students, achievement functioned as a source of validation that they could use to counter doubt. After finishing a capstone project that they had struggled to complete, one student reflected: “I felt very secure in myself and…felt like, yeah I do belong. You know? I do belong at this university, and I’m not only glad that they accepted me, but they should have.”

Many students also described boosts in their belonging after successfully presenting at conferences, suggesting that successful academic performance, especially when visible, served as a powerful tool for negotiating legitimacy. Success transformed belonging from a question into a claim that turned uncertainty into a sense of recognition.

*Comparisons With Peers.* Nonetheless, belonging was not determined only by internal feelings of success or failure. For many, it was molded through *comparisons with peers*: “when they look at this equation, they immediately understand the result and the relationship and how high or how low the resulting answer might be…and I’m like how do you just look at it and know?” While students are often unable to confirm peers’ actual abilities, perceptions can lead to feelings of inadequacy.

Another student echoed this unease: “sometimes I feel a little out of place because some people know a lot more about certain aspects.” Perceiving peers as more knowledgeable made it harder to feel legitimate or included, yet comparisons did not always result in a decreased sense of belonging.

For many, *shared* struggle became a source of validation:

*I feel a lot of people who look like me*,* or…people who are struggling with this feel like they don’t belong*,* and it’s just like a feeling*,* so I’ll be fine and I’m watching my peers do it*,* and I know my peers…feel the same way*,* like a little insecure about their ability to be a neuroscientist. So that helps*,* that confirms to me that it’s just a feeling*,* and I’ll watch one of the smartest people I know be like*,* ‘oh*,* I don’t think I’m good enough*,*’ and I’m like*,* you’re literally great. And so*,* it confirms in me that it’s just a feeling. And that helps.*

Here, seeing high-achieving peers express self-doubt served as a reminder that insecurity is not a unique experience, allowing a reframing of struggle as temporary rather than disqualifying. Peer comparisons therefore became a negotiation of belonging in relation to others. Although some encounters amplified feelings of inadequacy, others provided evidence that self-doubt was common and conquerable, enabling students to renegotiate legitimacy.

### Social dynamics

Students’ sense of belonging is deeply shaped by the social environments they navigate. The theme of social dynamics captures how students interpreted and responded to interpersonal and institutional cues of belonging. These categories go beyond internal feelings and perceptions as students enact negotiations of belonging through social interactions and structures that either affirm or invalidate their presence in academic spaces. Dynamics expressed through experiences of *social positioning*, *social reinforcement*, and *representation* play a critical role in whether students claim a rightful place in the academic community or are made to feel like outsiders within it. Additionally, *family dynamics* can either encourage or challenge their pursuit of belonging in academic programs and careers.

*Social Positioning.* One way in which social dynamics shaped students’ views of belonging was through social positioning: how they felt positioned among peers and within academic environments. This category captures how participants interpreted their standing in relation to others, often shaped by intersecting aspects of identity such as race, age, sexual orientation, gender, class, and transfer status. These perceived positions influenced how students navigated campus spaces and whether they felt entitled to occupy them.

Social positioning affected both access and belonging for some students by shaping the ways they were seen by others and how they negotiated their presence in academic and social contexts. For example, one student experienced tension based on age and life trajectory:

*He’s got Doctor next to his name*,* so he feels like that entitles him to deride people. And he knew nothing about me. Nothing about my background*,* you know*,* just because I’m an older student. Yeah*,* I’m an undergraduate student. But you know I’ve lived a life before I came [here]…that was definitely a moment where it’s like*,* wow*,* if there are people like this…Is this a department I want to be a part of…How many more people are there like this? If he’s achieved the success and hasn’t been corrected to this point…what does that mean for the department as a whole?*

This experience illustrates how older students can be socially positioned as outsiders. It is also an example of how institutional power can be embodied by faculty to reinforce ageist assumptions. Despite being demographically common at CCs (Bahr et al., 2021), nontraditional students may find themselves navigating academic cultures that fail to recognize or respect their life experiences. When students interpret faculty attitudes as reflective of institutional or disciplinary norms, it can also diminish their sense of trust and belonging on a broader scale, not just interpersonally.

While many older students were positioned as outsiders because they did not fit traditional expectations of what a college student “should” be, others encountered resistance for exceeding those expectations. For example, one student’s high performance and confidence did not lead to affirmation. Instead, it triggered social penalties arising from a disruption of others’ assumptions about their competence:

*I feel like people didn’t enjoy that I exceeded their expectations. It almost felt wrong…I did notice that some people felt the need to overcompensate…and pick on other things that I did to make me feel stupid…So I feel like there’s always this need to prove that I was dumb*,* since I couldn’t be dumb in other aspects.*

This experience touches on deeper psychological dynamics related to cognitive dissonance and expectation violations (Aubert-Teillaud et al., 2023). This student’s identity as a young Black woman left her vulnerable to harmful stereotypes on multiple occasions, enabling peers to view her through a deficit lens. While her academic success challenged this view, it led to attempts to belittle her in order to restore unfair deficit views, undermining her sense of belonging within the academic community. This example illustrates that even when students meet institutional expectations, they may still be required to negotiate exclusionary responses within the social environment, suggesting an unspoken social standard for “acceptable” expressions of competence.

*Social Reinforcement.* While social positioning focused on how students were seen and positioned by others, social reinforcement captures how feedback and social connection influenced the process of negotiation as students received feedback that reinforced or challenged their sense of belonging. Faculty were the most mentioned contributors to social reinforcement with spontaneous interactions boosting belonging:

*I asked [a faculty member] if he would mind meeting with me and my team…and he said yes and he also talked to me for like an hour and a half about what I wanted to do in the future…I think that was a moment where*,* not just amongst my peers*,* but like with someone working at the institution*,* I felt like I belonged.*

Similarly, receiving positive feedback from a faculty mentor sent powerful messages about contribution which, in turn, reinforced belonging: “I’m contributing to somebody else’s project and they’re saying it’s good…that’s the short-term dream right now is like, to contribute to other people’s projects in a meaningful way.” These examples show that attention and affirmation from faculty in students’ chosen fields can reinforce their sense of belonging.

Conversely, negative feedback made academic spaces unwelcoming: “I feel like [lab] is where I felt the most like I don’t belong here because I would do something wrong, and then I would get yelled at for it.” Rather than framing mistakes as part of the learning process, this experience framed error as a threat to belonging. Similarly, one student described being discouraged by faculty’s dismissive comments during lecture: “it was just like, ‘oh, you don’t really know this? Well too bad! We got to keep going,’ and I’m like woah, wait…I can’t absorb everything well.” These moments capture an absence or reversal of social reinforcement and how this can deepen feelings of inadequacy, reducing sense of belonging.

Peer relationships played an equally important role in reinforcing or disrupting belonging. For some students, connecting with peers with shared values, interests, or struggles reduced feelings of isolation: “I feel like relying a lot on people who I’ve met through the college has helped me feel more belonging because I know I’m not alone.” On the other hand, peer status policing deterred connections and created a toxic sense of competition:

*There’s this air of being afraid to tell people the grades I’m getting because then people won’t want to study with me…it often feels like people are testing each other to see if they can keep up or if they can actually perform.*

In such environments, academic competition becomes a barrier rather than a motivator, making it more difficult to build supportive peer networks that sustain belonging. The absence of peer connection and mutual understanding also introduced an additional dimension to social reinforcement (or the lack thereof) by intensifying feelings of isolation:

*I feel like there’s less room for people to collaborate in studying…that makes me feel like I don’t belong because…how is it that I’m going through the same thing as 300 plus people and I cannot find one person to study with?*

Although some students found connection and affirmation among peers, others were left navigating competitive and isolating environments that discouraged collaboration and led to a heightened sense of vulnerability, diminishing their sense of belonging. These examples show how social reinforcement itself is a form of negotiation. When students receive affirming feedback and find supportive networks, they are given opportunities to stabilize their sense of belonging. On the other hand, dismissive and competitive environments disrupt negotiations, leaving belonging unstable.

*Representation.* Beyond direct social interactions, students’ sense of belonging was also shaped by institutional signals. Particularly, students noted who was visible, whose experiences were centered, and who held positions of power and leadership. These forms of representation communicated who the institution was designed for and therefore who belonged there.

For many students, seeing others who shared aspects of their identity, culture, or experiences helped affirm that they, too, could take up space as their authentic selves: “when I’m around brown people, I can be authentically Black” and “when I’m around queer people, I feel seen and understood.” Demographics were mentioned as either affirming belonging: “I’m one-half Japanese and there is quite a large Asian population, so yeah” or preventing it: “There’s like 3% Black people…so I didn’t feel like I belonged in that sense.”

Belonging was also fostered through exposure to diversity at the institutional level which conveyed to some that a wide range of identities and perspectives could coexist:

*We’re all so different and unique and we all have very different backgrounds…It made me feel like I belonged a little more because… [undergraduates] all come from such different places. So*,* it’s like*,* it’s ok not to have the exact same upbringing or experiences.*

While this sentiment does not reflect shared identities or experiences, it illustrates how representation can operate through the normalization of difference by creating a space where individuality and diversity are expected.

Interestingly, students who were accustomed to more diverse environments perceived a loss of diversity after transferring, complicating their development of a sense of belonging: “Jumping to here where it’s not as diverse, I really had to figure out how to find community.” This reflection highlights an important distinction: while institutional metrics may show diversity overall, that diversity may not always include the specific forms of representation that matter to individual students.

For some, a perceived lack of shared identities or experiences created a sense of isolation and invisibility. Age was a recurring source of tension for returning students which prevented them from integrating with their peers:

*Regardless of racial background or gender background*,* it’s easier to get people of those different backgrounds to kind of naturally fit together if they’re of the same age group and have largely the same experiences…it’s harder to bring someone in that’s old enough to be your dad.*

For one student, the divide was widened by their international status and geopolitical context:

*When I was in community college*,* there was a lot happening in my country…I couldn’t talk to my parents for 2 months…they shut down the internet…and the government killed people in the street…At that time*,* I felt like my body was in the US but my mind was somewhere else…when I tried to explain what happened in my country—they don’t listen to the news or they were talking about the weather. Even when I asked for a few days off because I couldn’t even stop crying*,* my manager said*,* “no*,* just don’t think about it and work.” Yeah*,* I didn’t have a sense of belonging there at all.*

This student progressively withdrew from peer interactions as they continued in their academic journey, illustrating how belonging can be disrupted by more than visible differences, including one’s lived reality. The absence of empathy and awareness from both peers and institutional representatives exacerbated their sense of isolation as their need for understanding and flexibility were dismissed. Furthermore, their interactions revealed a campus culture unprepared to support students carrying geopolitical trauma. In this case, the barrier to belonging was not due to demographic differences alone but also the absence of shared meaning and understanding which left the student feeling alienated and emotionally unsupported.

These examples illustrate how negotiations are tied to representation as students constantly interpret who and what is made visible. These interpretations are used to negotiate internally about what institutional signals mean for their own legitimacy, authenticity, and ability to occupy space.

*Family Dynamics.* Outside of the academic environment, family dynamics played an important role in how students thought about their academic futures. For students from underprivileged backgrounds, the pressure to prioritize financial stability often conflicted with long-term academic goals:

*Having to say*,* “yeah*,* I want to go to a PhD program to make 40k a year.” And they’re like*,* “why…you should just go and work at McDonald’s…you can make like 23 [dollars per hour] now”…I feel like in that way*,* academics has kind of created some sort of rift in my belonging back home.*

This students’ reflection shows how academic ambition can disrupt connections to home and family or community. For some, especially those from low socioeconomic backgrounds, the pursuit of higher education introduced a tension between belonging in academia and maintaining familial connections through shared values and meeting expectations. In these cases, belonging was not only about institutional or peer acceptance, it was also about coherence across students’ life spheres. The values closest to them, often stemming from cultural and familial commitments, complicated their academic plans and at times challenged their desire to belong in academic spaces and pursue academic careers.

Potentially informed by their cultural backgrounds and values, students also expressed concerns about potential misalignments with the prevailing norms of their chosen discipline:

*I’ve thought about if I wanted to be in the field. At times I felt like it was really competitive and it’s sort of the capitalistic grind…so I had times wondering if that’s what I wanted to do.*

In these instances, the issue was not exclusion, but an internal conflict about whether the culture of the discipline resonated with the students’ sense of purpose and identity which may reflect or be closely tied to their family values. Negotiations of belonging, therefore, crossed domains as students weighed the costs of aligning with academic or disciplinary norms against the risk of distancing themselves from family or cultural values.

### Institutional conditions

On a broader scale, students expressed concerns about fairness. Particularly, as they observed how easily privileged and “traditional” students navigated the institution. This theme captures students’ experiences with rigid academic and social expectations, as well as the structural barriers and exclusionary practices embedded in institutional systems. Institutional conditions represent the broader environments within which students negotiated belonging. These often perpetuated inequality by privileging those who fit expected norms while forcing others to provide justifications for their presence.

*Equal Expectations in Unequal Conditions.* This category captures the tension experienced when students realized that their institution’s universal standards did not account for differences in background, resources, life circumstances, or preparation and instead assumed a “level playing field” that did not exist. This experience began at the CC level for one student as they struggled to find opportunities to support their academic aspirations:

*The whole time I was in community college*,* I felt inadequate because I couldn’t find research experience…you get to university and you have these professors telling you…’oh*,* yeah*,* just chill…you’re competitive as it is*,*’ but I wish there was an aspect that was more pertinent to what is actually going on…keeping in mind that we’re transfers*.

Importantly, this student’s feelings of inadequacy did not stem from a lack of motivation but from a lack of access to research, mentorship, and networking in the CC context. These inequities did not disappear after the student transferred; instead, they became invisible to faculty who assumed all students had access to equal opportunities and preparation, reinforcing the myth of equality. The professor’s reassurance was likely meant to ease pressure, but instead inadvertently dismissed the structural barriers that shaped the student’s path. In this case, the student is not asking for lowered standards but for acknowledgement that transfer students may have distinct needs. This quote highlights a desire for responsiveness over expected assimilation and highlights how meritocratic ideology continues to be reproduced by faculty in higher education through the obscuring of systemic inequalities.

The sense of invisibility and dismissal was compounded when students observed inequities not only in faculty expectations but also in how peers leveraged unacknowledged advantages. Students experienced feelings of defeat as they witnessed peers succeeding through unspoken advantage: “just the day before, they were popping molly…yet they can pay a private tutor and get an A in the class.” This student’s reflection emphasizes how privileged peers may access resources that mask underperformance, allowing them to maintain academic performance despite disengagement. Meanwhile, transfer students and those from less privileged backgrounds often face structural barriers that limit their access to support and their visibility within academic spaces. This contrast highlights how wealth can shield some students from the academic consequences while others must proceed without comparable safety nets.

Recognizing these disparities can erode students’ sense of belonging by reinforcing the belief that the institution is designed for those whose advantages are assumed and unquestioned. Such advantages are not always financial. Some students experienced a sense of disadvantage rooted in uncontrollable life circumstances that disrupted their preparation long before entering college. One student reflected: “They have the study habits that I didn’t necessarily get to form yet…just because from the age of 14 and on, my life was always so unpredictable.” This reflection reveals how students may interpret their struggle as a consequence of disrupted stability and support rather than a lack of ability. The result: a sense of loss when they recognize that others were better positioned to succeed.

Similarly, chronic health conditions contributed to feelings of alienation. One student described the emotional toll of being held to the same expectations as peers while managing health challenges:

*It’s really important that my health comes first because I can’t do well in other aspects if that doesn’t happen*,* but I didn’t always feel like that was honestly encouraged…I felt like my work was supposed to come first in a lot of situations.*

When students who have faced long-term adversity are held to the same standards as those with more stable lives, they may feel unfairly evaluated and ultimately misplaced within academic environments. Without institutional recognition of their unequal conditions, students are left to negotiate their belonging under unequal terms that leave them questioning their legitimacy and right to pursue higher education.

*Structural Inequality.* While some encountered academic barriers based on personal life circumstances, others pointed out systemic inequalities entrenched within institutional structures. These inequalities consistently privileged certain student populations over others, regardless of individual effort. Structural dynamics went beyond academic expectations, shaping access, opportunity, and visibility in ways that marginalized transfer students and underrepresented groups.

Transfer students often describe unacknowledged “time pressure” (Duncheon et al., 2025), feeling rushed to meet academic milestones. Participants in this study were no exception: “You’re expected to be at the same level as [4-year students].” They also expressed feeling overlooked for scholarships and research opportunities based on unrealistic eligibility criteria: “[transfer students] are not really considered for a lot of things…even with scholarships…you have to have a minimum of this many extracurriculars or this many years of research…that’s just not realistic as a transfer.”

In addition to feeling excluded from opportunities, students also noted how research spaces themselves were culturally misaligned with their identities and values: “research wasn’t really built for underrepresented minorities…it’s very obvious with the culture and atmosphere…a lot of identities can get suppressed.” Moreover, research was made even less accessible and less appealing by the expectation of unpaid labor: “I think of the fact that a lot of people work for free at labs…I’m like, ‘why would I work somewhere for free?’

The mental and emotional toll of navigating these inequities added to the mental burden of marginalized students, who came to see their own struggles as out of the norm: “I feel like there’s a larger population of students who had ‘normal upbringing’ / ‘privileged upbringing’ in terms of finance.”

Together, these accounts reveal how institutional structures normalize a specific academic trajectory that often aligns with financial stability, uninterrupted development, and early academic socialization. For students who fall outside of these norms, the burden is not only to prevail academically but to negotiate belonging under conditions that require them to adapt. Unlike personal hardship alone, this category captures a deeper logic of exclusion embedded in how opportunity is structured, rewarded, and recognized in academic institutions. This systemic misalignment reinforces social hierarchies and conveys who truly belongs and who must continually negotiate, adapt and earn their place.

## Discussion

### Limitations

In this paper, we have described a theoretical framework for understanding a sense of belonging informed by the experiences of transfer students interested in neuroscience pathways. While these insights have the potential to deepen our understanding of belonging as a negotiated process, this study is limited by its small sample size as well as its context. Participants were part of a structured support program that provided them with financial assistance and mentorship. The program provided access to resources and forms of recognition not typically available to transfer students. Therefore, participants’ experiences may reflect a best-case scenario rather than typical conditions. Accordingly, we do not claim that these findings are fully generalizable to all STEM transfer students. However, because participants were situated within a relatively well-resourced context, the factors they identified may highlight aspects of belonging that are particularly susceptible to targeted interventions.

Notably, participants did not frequently attribute their sense of belonging to program-specific elements. Instead, they more frequently emphasized intrinsic interest in their field and personally meaningful disciplinary engagement. One possible interpretation of this finding is that students’ orientations toward and constructions of belonging may be shaped more strongly by how they identify with their discipline rather than by institutional involvement. Further research is needed to examine how these dynamics operate in less-supported contexts.

### Integration with prior studies

Participants described their sense of belonging as a negotiated, context-dependent process rather than as a static feeling. Many of their critical experiences involved descriptions of academic spaces and social interactions, supporting prior conceptualizations of belonging as shaped by social, physical, and disciplinary environments (Allen et al., 2022; Strayhorn, 2019; Vaccaro & Newman, 2022).

Our findings suggest that belonging is an interpretive process rooted in students’ prior experiences, orientations, and survival strategies. While institutional environments and relationships matter, students’ sense of belonging is not solely determined by these conditions. A key contribution of this study is an individual’s orientation towards belonging which emerged as a foundational perspective through which students either deemphasized or stressed the importance of belonging. Orientation toward belonging captures students’ beliefs about how belonging is built as well as the importance of their intentions to build it, aligning with Mahar’s and colleagues’ (2013) self-determination dimension of belonging. Though the unaware orientation was the least common among participants, its presence suggests that, for some, belonging and participation are experienced as assumed rather than as something that requires negotiation. This orientation may be more prevalent among those whose identities align more closely with dominant student narratives of who belongs in STEM, which may help explain why it was less common among CC transfer students.

This interpretation aligns with prior research showing that male and White students often report higher levels of belonging in STEM contexts than women and students from racially minoritized or underrepresented groups (Flores et al., 2024; LaDue et al., 2024; Steele et al., 2020). Taken together, these findings and prior research (e.g., Vaccaro & Newman, 2016) suggest that students whose identities are more closely aligned with dominant disciplinary norms may be less likely to experience belonging as something that must be negotiated or intentionally built.

Furthermore, students’ narratives illustrate belonging as a transitional and time-dependent process informed by prior educational and non-academic experiences, consistent with Dost’s (2026) conceptualization of belonging as developmental. However, transfer did not automatically prompt students to seek belonging within their transfer institution. Instead, students varied in how they interpreted the importance of belonging in new academic contexts, including what groups they sought to connect with and the extent to which they pursued belonging within the university community. These findings suggest that belonging is continuously renegotiated through students’ ongoing experiences and in-the-moment interpretations.

Participants’ negotiations of belonging also revealed distinctions between institutional and disciplinary forms of belonging. When characterizing disciplinary belonging, students often described internal factors such as personal interest, curiosity, and passion toward their chosen discipline as signals of belonging, even in the absence of social connectedness or acceptance. Furthermore, belonging within their field was not contingent on experiencing a strong sense of belonging among peers or within the broader institution, highlighting a distinction between field-specific and institutional or social forms of belonging. These findings suggest that students may build a sense of belonging to their chosen discipline through intellectual engagement and personal identification with the field, even when their integration into disciplinary communities or environments remain limited.

### Implications

These findings have important implications for how institutions can approach supporting students’ sense of belonging. In particular, curricular structures that allow students to explore their interests, connect coursework to broader disciplinary purposes, and engage in inquiry-based or research-oriented experiences can provide important avenues for students to affirm their place in neuroscience and STEM fields more broadly. Moreover, institutions should recognize that students do not build a sense of belonging in the same ways or through the same communities. Efforts to support belonging may therefore be most effective when they create multiple opportunities for students to connect, feel recognized, and participate within their chosen disciplines rather than treating the broader institution as the primary anchor for students’ sense of belonging. This kind of engagement need not be implemented via a funded program like the one our students participated in; it can also be supported through pedagogical practices such as project-based, community-engaged, or civic learning.

## Conclusion

This study adds to the literature on sense of belonging in STEM by theorizing belonging as a negotiated process conducted through students’ interpretations of personal, structural, and social cues. Students initiate the process by asserting an orientation toward belonging and proceed by evaluating their perceived legitimacy within academic spaces which are signaled by both internal feelings and interpretations (e.g., intrinsic motivation, perceived academic performance) and external feedback (e.g., social dynamics and institutional conditions). This model advances existing frameworks by centering the experiences of STEM transfer students and emphasizing their individual interpretations of belonging in the face of structural inequality. While social connection is often cited as a key factor in persistence, this study emphasizes intrinsic values such as personal interest which also plays a key role in students’ negotiations of belonging.

For institutions aiming to foster belonging among underrepresented CC transfer students, it is not enough to promote inclusion alone. Academic support must include genuine academic exploration that enables students to discover and pursue personal interests that can sustain long-term engagement. Institutions must also address the structural conditions under which belonging is unequally granted, earned, or withheld. More broadly, this study highlights the importance of understanding belonging as a process that unfolds across contexts rather than within a single institution or environment. For transfer students, belonging is continually negotiated as they carry prior experiences, identities, and aspirations into new academic experiences.

## Data Availability

The datasets generated and/or analyzed during the current study are not publicly available due to the sensitive nature of the data and need to protect participant confidentiality but are available from the corresponding author on reasonable request.Ethics Approval and Consent to Participate: This study was reviewed by the University of California, San Diego Institutional Review Board and determined to be exempt from formal ethics approval. Participation was voluntary, and consent was verbalized during individual interviews. Participants voluntarily shared their experiences with the understanding that their data would be anonymized for research purposes.

## Footnotes

1 We use the term “Latine” instead of “Latinx” because it comes directly from the Spanish-speaking community and therefore integrates with Spanish grammar, making it easier to pronounce and more inclusive for Spanish-speakers.

## Abbreviations

AANAPISI: Asian American and Native American Pacific Islander Serving Institution
CC: Community College
HSI: Hispanic Serving Institution
STEM: Science, technology, engineering, and mathematics fields
